# Diethylstilbestrol Modifies the Structure of Model Membranes and Is Localized Close to the First Carbons of the Fatty Acyl Chains

**DOI:** 10.3390/biom11020220

**Published:** 2021-02-04

**Authors:** Alessio Ausili, Inés Rodríguez-González, Alejandro Torrecillas, José A. Teruel, Juan C. Gómez-Fernández

**Affiliations:** Departamento de Bioquímica y Biología Molecular “A”, Facultad de Veterinaria, Regional Campus of International Excellence “Campus Mare Nostrum”, Universidad de Murcia, Apartado de Correos 4021, E-30080 Murcia, Spain; aausili@um.es (A.A.); ines.rodriguez@um.es (I.R.-G.); alexts@um.es (A.T.); teruel@um.es (J.A.T.)

**Keywords:** diethylstilbestrol, membranes, ^31^P-NMR, ^1^H-NOESY-MAS-NMR, DSC, wide-angle X-ray diffraction (WAXD), SAXD

## Abstract

The synthetic estrogen diethylstilbestrol (DES) is used to treat metastatic carcinomas and prostate cancer. We studied its interaction with membranes and its localization to understand its mechanism of action and side-effects. We used differential scanning calorimetry (DSC) showing that DES fluidized the membrane and has poor solubility in DMPC (1,2-dimyristoyl-*sn*-glycero-3-phosphocholine) in the fluid state. Using small-angle X-ray diffraction (SAXD), it was observed that DES increased the thickness of the water layer between phospholipid membranes, indicating effects on the membrane surface. DSC, X-ray diffraction, and ^31^P-NMR spectroscopy were used to study the effect of DES on the L_α_-to-H_II_ phase transition, and it was observed that negative curvature of the membrane is promoted by DES, and this effect may be significant to understand its action on membrane enzymes. Using the ^1^H-NOESY-NMR-MAS technique, cross-relaxation rates for different protons of DES with POPC (1-palmitoyl-2-oleoyl-*sn*-glycero-3-phosphocholine) protons were calculated, suggesting that the most likely location of DES in the membrane is with the main axis parallel to the surface and close to the first carbons of the fatty acyl chains of POPC. Molecular dynamics simulations were in close agreements with the experimental results regarding the location of DES in phospholipids bilayers.

## 1. Introduction

**Figure 1:**
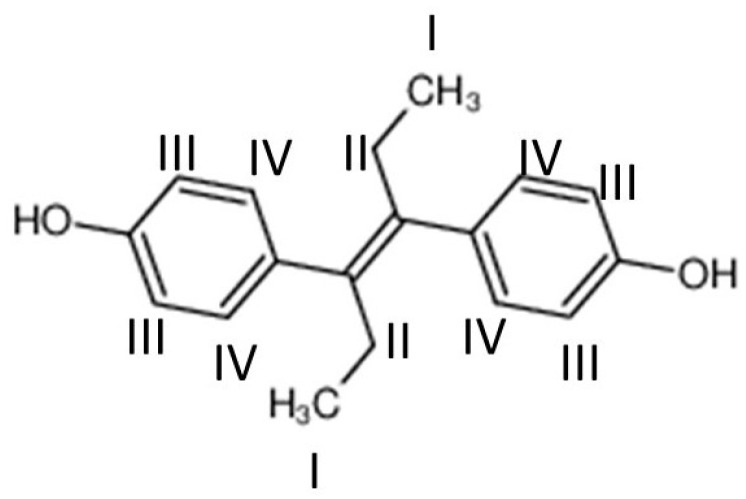
Structure of diethylstilbestrol (DES). Protons studied by ^1^H-NMR are labeled.

Diethylstilbestrol (DES) is a synthetic form of estrogen, a female hormone. Its name, established by the International Union for Pure and Applied Chemistry IUPAC, is 4-[(*E*)-4-(4-hydroxyphenyl)hex-3-en-3-yl]phenol (Figure 1).

DES consists of a drug with antiestrogenic and antiandrogenic effects, used to treat metastatic carcinomas and prostate cancer. It was widely used during some decades (1940–1970) to prevent spontaneous abortions, but it was later found that DES causes very important toxicological effects to the children exposed in the uterus to this substance, such as an increase in the risk of cancer and abnormalities in the uterus of daughters, and these effects may be seen even in the third generation [1]. Currently, this synthetic estrogen is used in rare cases, due to the existence of other drugs which cause fewer secondary effects, since DES is related to the appearance of carcinomas of the vagina and uterus in those women whose mothers received DES during their pregnancy [2,3]. DES is also an endocrine disruptor with obesogenic effects [4].

DES is an amphipathic molecule with membrane affinity, and it has been shown that it may interact with membrane enzymes such as H^+^-ATPases of the P-, V-, and F- types [5]. For example, it inhibits the H^+^-ATPase of rat liver mitochondria, interfering with the proton translocation activity of the F_o_ subunits [6,7]. It was also observed that DES may inhibit the Ca^2+^-ATPase transport system of the sarcoplasmic reticulum after insertion into the membrane, affecting the Ca^2+^-binding sites [8]. These inhibitory effects of DES indicate that some of its biological effects may be associated with its insertion into the membrane.

Most interesting is the fact that the effects of DES may be mediated by membrane estrogen receptors. It has been shown that mice genetically engineered to lack membrane estrogen receptors do not suffer pathologies associated with exposure to DES [9].

Keeping in mind the clear association of the biological effects of DES with its insertion into membranes, it is interesting to study its interaction with model membranes and its membrane location. Given its molecular structure, it should be considered whether this molecule may alter the properties of the membranes, since it is possible that this modification might be one of the causes of its biological effects. The diverse biological activities of DES have not yet been explained from a molecular point of view. One may expect that its mechanism of action could be its interaction with hormone (estrogen) receptors since it is a synthetic structural analogue of these hormones. However, although it is true that DES could interact with the receptors of these hormones, it may interact also with membranes and may alter the activity of membrane enzymes such as H^+^-ATPases of the P-, V-, and F- types [5]. It is then interesting to characterize the way in which DES modulates the membrane to fully understand its molecular mechanism of action.

Therefore, in this work, we studied the interaction of DES with model membranes of DMPC (1,2-dimyristoyl-*sn*-glycero-3-phosphocholine) to study its modulation of the membrane gel-to-fluid transition and membrane dimensions, of DEPE (1,2-dielaidoyl-*sn*-glycero-3-phosphoethanolamine) to study its influence on membrane curvature, and of POPC (1-palmitoyl-2-oleoyl-*sn*-glycero-3-phosphocholine) to study its location in the membrane.

## 2. Materials and Methods

### 2.1. Materials

1,2-Dimyristoyl-*sn*-glycero-3-phosphocholine (DMPC), 1,2-dielaidoyl-*sn*-glycero-3-phosphoethanolamine (DEPE), and 1-palmitoyl-2-oleoyl-*sn*-glycero-3-phosphocholine (POPC) were produced by Avanti Polar Lipids (Alabaster, AL, USA) and purchased from Sigma-Aldrich (Madrid, Spain).

Diethylstilbestrol (DES) and all other reagents and solvents used in the experiments were acquired from Sigma-Aldrich (Madrid, Spain).

### 2.2. Sample Preparation

For all experiments and techniques, multilamellar vesicles (MLVs) were used as models of study. The phospholipids used to generate the samples were DMPC, POPC, and DEPE. Phospholipid dispersions with and without DES were prepared by hydrating the required amounts of DMPC, POPC, or DEPE and DES in 10 mM 2-[4-(2-hydroxyethyl)piperazin-1-yl]ethanesulfonic acid (HEPES), 100 mM NaCl buffer, 0.2 mM 2,2’,2’’,2’’’-(Ethane-1,2-diyldinitrilo)tetraacetic acid (EDTA), pH 7.5. The desired amounts of phospholipids and DES (depending on the type of experiment) dissolved in chloroform/methanol solutions (2:1) were mixed to obtain the desired concentrations. The solvents were carefully evaporated under a flow of nitrogen, and the residual traces of solvent were removed via high-vacuum drying for at least 3 h. The buffer was added to the dried samples and vortexed until a homogeneous suspension of phospholipids was obtained, always keeping the temperature above the gel to liquid-crystalline phase transition temperature of the phospholipids.

The concentration of the phospholipids was determined by the Böttcher method [10]. The molar ratios of phospholipid/DES were checked by using ^1^H-MAS-NMR.

### 2.3. Differential Scanning Calorimetry (DSC)

DSC samples were prepared as described above using 2.2 µmol of phospholipids in the absence and presence of DES. The phospholipid/compound molar ratios were 60:1, 30:1, 15:1, 7:1, 3:1, and 1:1 for DMPC/DES mixtures and 80:1, 40:1, 20:1, and 10:1 for DEPE/DES mixtures. To prepare the MLVs, the samples were hydrated in 1.5 mL of buffer to obtain a phospholipidic concentration of about 1 mg/mL. Measurements were performed with a high-resolution MicroCal MC-2 microcalorimeter (Microcal, Northampton, MA, USA) using in the reference cell the same buffer employed to prepare the vesicles. Samples and reference were degassed for 10 min before being loaded into the microcalorimeter. The temperature range of the scans was between 10 and 40 °C for DMPC samples, and up to 70 °C for DEPE in order to observe the formation of the hexagonal phase and the effect of DES on it, while the scanning rate was 1 °C/min for all samples. Three successive heating scans were recorded for each sample to guarantee scan-to-scan reproducibility, and the last scan was utilized for data analysis, which was performed with the Microcal Origin 5.0 software (OriginLab, Northampton, MA, USA). A buffer vs. buffer thermogram was subtracted from all sample thermograms prior to data processing. The parameters calculated from the final thermograms were onset and end transition temperature.

### 2.4. X-Ray Diffraction

Small-angle (SAXD) and wide-angle (WAXD) X-ray diffraction measurements were simultaneously performed using a modified Kratky compact camera (MBraun-Graz-Optical Systems, Graz, Austria), using two coupled linear position sensitive detectors (PSD, MBraun, Garching, Germany) to monitor the s-ranges (s = 2 sin θ/λ, 2θ = scattering angle, λ = 1.54 Å) of 0.0075–0.07 and 0.20–0.29 Å^−1^, respectively. Nickel-filtered Cu KR X-rays were generated by a Philips PW3830 X-ray generator operating at 50 kV and 30 mA. The detector position was calibrated by using Ag-stearate (small-angle region, d-spacing at 48.8 Å) and lupolen (wide angle region, d-spacing at 4.12 Å) as reference materials. Pellets of the samples were placed in a steel holder with cellophane windows. X-ray diffraction profiles were obtained for 10 min exposure times. Background-corrected SAXS data were analyzed using the GAP (global analysis program) written by Georg Pabst and obtained from the author [11,12]. This program allowed retrieving the membrane thickness, d*B* = 2(z_H_ + 2σ_H_), from a full *q*-range analysis of the SAXS patterns [13]. The parameters z_H_ and σ_H_ are the position and width, respectively, of the Gaussian used to describe the electron-dense headgroup regions within the electron density model. The water layer was then given by d*w* = *d* − d*B*.

### 2.5. ^31^P-NMR

First, 25 mg of DEPE in the presence and in the absence of the desired amount of DES was used in all the cases to obtain good-quality spectra. Dried samples were resuspended in 50 μL of 10 mM HEPES, pH 7.4, and multilamellar vesicles (MLVs) were prepared as described above.

^31^P-NMR spectra were obtained using a Bruker Avance 600 instrument (Bruker, Etlingen, Germany) operating at of 242.9 MHz. To obtain the ^31^P spectra, 5 μs pulses were used with gated decoupling ^1^H. The samples were placed into 5 mm outer diameter OD NMR glass tubes, and the spectra were recorded at different temperatures. All spectra were obtained in the presence of a gated broad-band proton decoupling (5 W input power during acquisition time), and accumulated free inductive decays were obtained from up to 8000 scans. A spectral width of 48,536 Hz, a memory of 48,536 data points, a 2 s interpulse time, and a 90° radio frequency pulse (11 μs) were used with inverse gated decoupling ^1^H. Prior to Fourier transformation, an exponential multiplication was applied, resulting in a 100 Hz line broadening.

### 2.6. ^1^H-NOESY-MAS-NMR

To prepare samples, 25 mg of POPC and the desired amount of DES were dissolved in a chloroform/methanol mixture (2:1 *v*/*v*) and desiccated under vacuum; then, multilamellar vesicles were formed by adding deuterated water. NMR measurements were carried out on a Bruker Avance 600 spectrometer, operating at 600 MHz, equipped with an HRMAS probe and using ZrO_2_ of 4 mm BL4 rotors with Kel-f BL4 caps at 25 °C. The spin rate was 8 kHz; data points were 1024 from 16 scans, and the spectral width was 20 ppm. The relaxation delay was 3.5 s and the mixing time was 300 ms. Two-dimensional NOESY experiments were acquired using 90° pulses of 5.5 µs. Data were processed using TopSpin 2.1 software (Bruker, Etlingen, Germany), supplied by Bruker.

The location probability was estimated from data obtained at a mixing time of 300 ms, according to [14].

$$\sigma_{ij}=\frac{\left(A_{ij}\left(t_{m}\right)\right)}{\left(A_{jj}\left(t_{m}\right)\right).\;t_{m}}$$

where *σ_ij_* is the cross-relaxation rate, *A_ij_* is the cross-peak volume, *A_jj_* is the diagonal peak volume, and *t_m_* is the mixing time of the NOESY spectrum. If has been discussed [15] that a single mixing time MAS-NOESY experiment is sufficient for characterizing intermolecular interactions in membranes as supported by previous extensive work [14,16].

### 2.7. Molecular Dynamics Simulations

The molecular structure of DES was obtained from the PubChem Substance and Compound database [17] through the unique chemical structure identifier CID 448537. Molecular dynamics simulations were done with GROMACS 5.0.7 software [18] and the Gromos 53A6 force field [19,20] . The topology file for POPC was described by Poger et al. [21,22]. The topology file for DES was obtained using the Automated Topology Builder and Repository [23,24]. Each membrane leaflet containing 64 POPC molecules, 2500 water molecules simulated by the single point charge model, and 13 DES molecules was built using Packmol software [25]. Other conditions were used as previously described [26,27,28,29]. Equilibration was done in the isothermal–isobaric ensemble at 298 K constant average temperature for 100 ns using the V-rescale temperature coupling method [30]. Pressure was controlled semi-isotropically using the Berendsen pressure coupling method [26] . Equilibration was followed by production runs of 100 ns using the Parrinello–Rahman barostat [29]. Analysis of the last 60 ns from the trajectory of the production run was done using GROMACS analysis tools. Molecular dynamics calculations were carried out at the Computational Service of the University of Murcia (Murcia, Spain).

## 3. Results

### 3.1. DSC Study of the Interaction of DES with DMPC

The technique of differential scanning calorimetry (DSC) is very adequate for studying the interaction of substances with a membrane. It allows monitoring the gel-to-fluid phase transition of the model membrane as the temperature is modified, with the heat capacity of the system at constant pressure (Cp) being measured as a function of the temperature. The obtained thermograms permit the characterization of the transition temperature which could decrease if the membrane is fluidized by the interaction with an added molecule, the increase in enthalpy (ΔH), which is directly proportional to the area under the transition trace, and the width of the transition, which is indicative of the cooperativity of the transition (a narrower peak denotes greater cooperativity).

**Figure 2:**
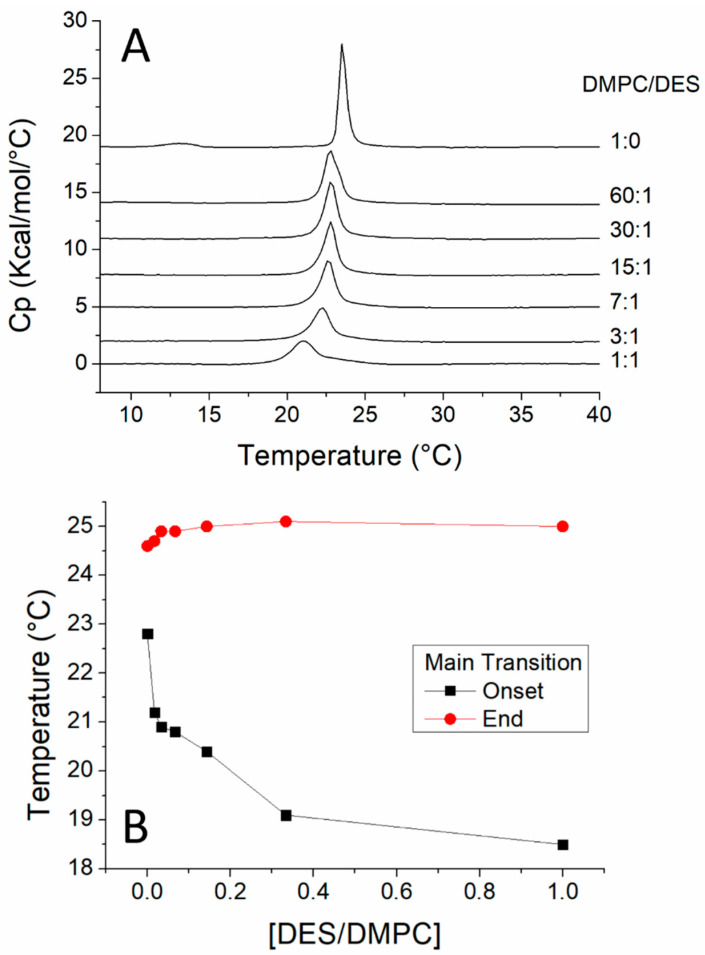
(**A**). Thermograms obtained by using differential scanning for mixtures of DMPC (1,2-dimyristoyl-*sn*-glycero-3-phosphocholine) and DES. The phospholipid/DES molar ratios are given on the traces. (**B**) Plot of DES/DMPC molar ratios versus temperature, on which the temperatures corresponding to onset and end of phase transitions are plotted.

We observed that DES decreased the transition temperature (Figure 2A) and the heat capacity of DMPC (Table 1) as these effects increased with increasing concentrations of DES. Small concentrations of DES such as 60:1 DMPC/DES (molar ratio) already induced the disappearance of the pretransition observed in pure DMPC starting at 11 °C. The effect on the pretransition is a characteristic of intrinsic molecules. The effect of DES on the phase transition can also be observed through the decrease in ΔH (Table 1) since the increase in DES concentration was accompanied by a decrease in the size of the peak originated in the thermogram by the phase transition. This alteration in the phase transition can be explained by the interposition of DES molecules between the acyl chains of DMPC molecules, altering the packing of these chains with the introduction of disorder. The other effect was a widening of the transition peak, indicating a decrease in the cooperativity of the transition. A partial phase diagram plotting the temperatures of beginning and completion of the main transition (Figure 2B) shows the temperatures of onset and completion of the thermograms, from which a partial phase diagram could be elaborated. Although the beginning and end transition temperatures had a reversal effect with regard to the concentrations of DES, they remained constant at concentrations of 30:1 (DMPC/DES molar ratio and higher). This indicates a low solubility of DES in DMPC with a fluid immiscibility.

### 3.2. DSC Study of the Interaction of DES with DEPE

The interaction of intrinsic molecules with DEPE is a test widely used to study the propensity of these molecules to favor negative curvature in the phospholipid membrane and nonlamellar phases, as it has been extensively studied [31].

We use the term intrinsic molecules in this work to denominate small molecules that are inserted in the membrane and have a hydrophobic interaction with the acyl chains of the phospholipids. In a broad sense, integral proteins and even phospholipids are also intrinsic molecules.

**Figure 3:**
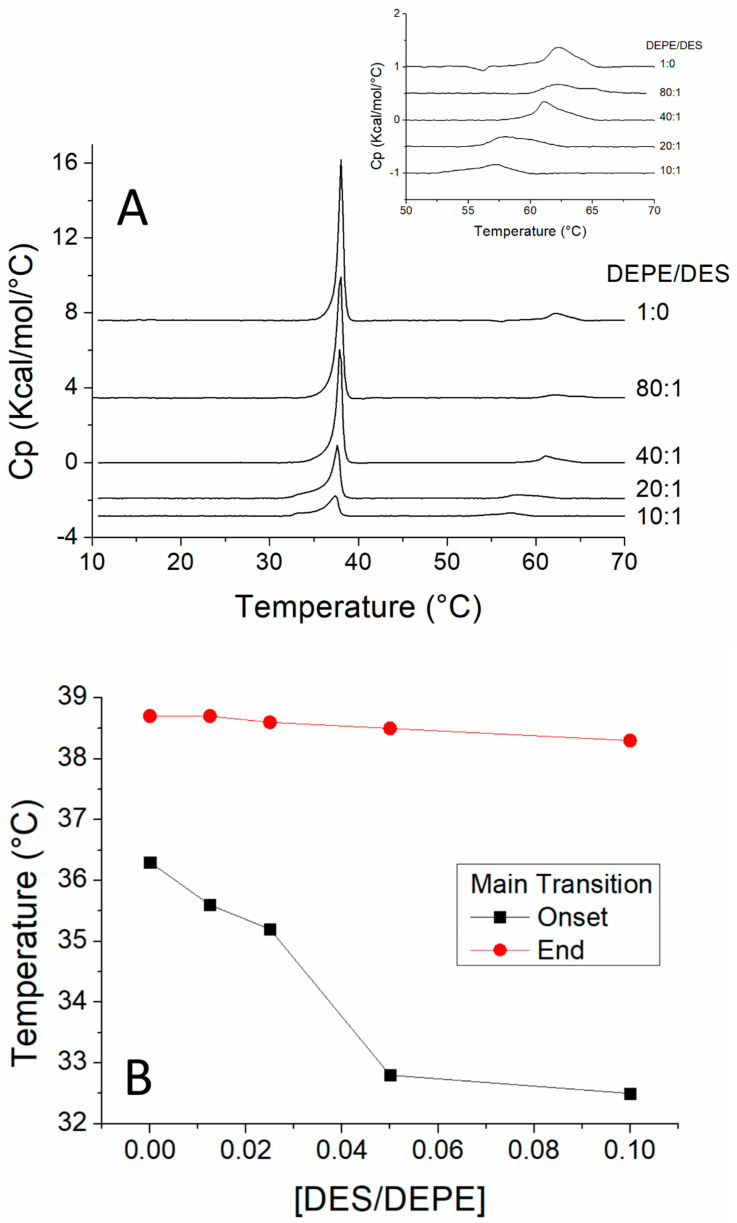
(**A**) Heating scan thermograms, obtained by using differential scanning for mixtures of DEPE and DES. The phospholipid/DES molar ratios are given on the traces. The transition of L_α_ to H_II_ phases (T_H_ is the onset of this transition) is amplified in the insert. (**B**) Plot of DES/DEPE molar ratios versus temperature on which the temperatures corresponding to onset and end of phase transitions are plotted.

When studied by DSC in heating scans, pure DEPE showed a first transition from L_β_ (lamellar gel) to L_α_ (lamellar liquid crystalline) with T_c_ at 35 °C and a second transition from L_α_ to H_II_ (inverted hexagonal) with T_c_ at 62 °C (Figure 3). In the presence of increasing concentrations of DES, the T_c_ of the thermotropic phase transition from L_β_ to L_α_ of DEPE membranes gradually decreased, as did ΔH, evidenced by the reduction in size of the transition endotherm (Table 1), as similarly observed for the DMPC/DES system. It should also be noted that the temperature of completion of this phase transition did not change upon an increase in concentration of DES indicating an immiscibility in the fluid phase with DEPE. An important observation is that T_H_, i.e., the beginning of the transition from L_α_ to H_II_, decreased with increasing concentrations of DES (Figure 3 insert), indicating that DES favors a negative curvature of the membrane.

### 3.3. X-Ray Diffraction

**Figure 4:**
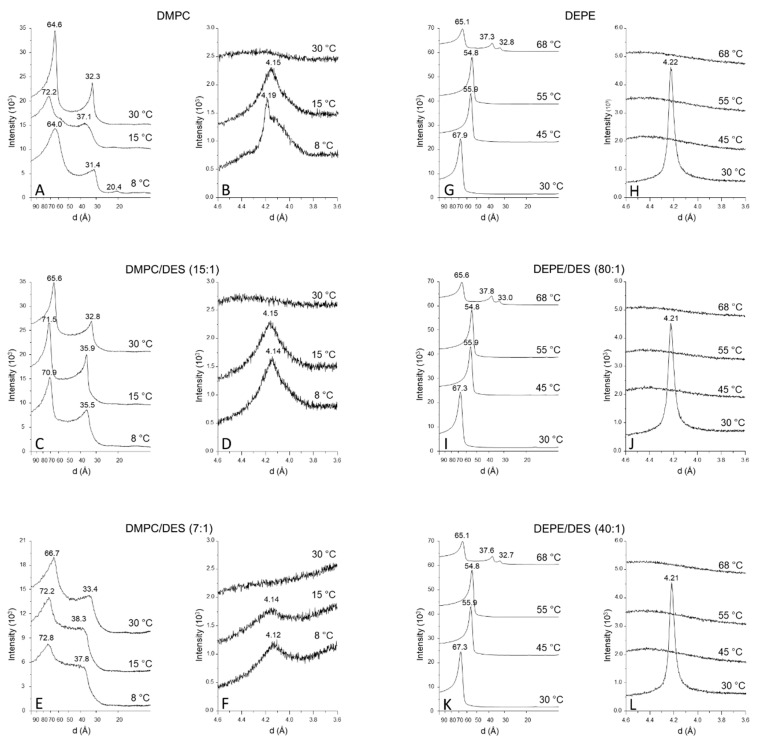
Small-angle (SADX; **A**,**C**,**E**,**G**,**I** and **K**) and wide-angle (WADX; **B**,**D**,**F**,**H**,**J** and **L**) X-ray diffraction profiles of DMPC/DES mixtures: pure DMPC (**A**,**B**); DMPC/DES 15:1, molar ratio (**C**,**D**); DMPC/DES 7:1, molar ratio (**E**,**F**); pure DEPE (**G**,**H**) DEPE/DES 80:1, molar ratio (**I**,**J**); DEPE/DES 40:1, molar ratio (**K**,**L**). Temperatures are shown on the traces. Values corresponding to d-spacings in Å are also given on the diffractograms.

The SAXD diffractogram of pure DMPC shows the well-described pattern for multilamellar vesicles with d-spacings of first order and later following the ratios 1:1/2:1/3 [32,33,34,35]. This is the case at 8 °C (Figure 4A) with d-spacings giving approximate ratios of 64.03/31.41/20.40 Å. Similarly, at 15 °C, the observed d-spacings appear at 72.18/37.12 Å and, at 30 °C, they appear at 64.55/32.30 Å. The results obtained from the WAXD permit deducing the packing of the phospholipids in the bilayer. Figure 4B shows that, at 8 °C (below the pretransition), pure DMPC shows a sharp peak at 4.19 Å and a broad shoulder at 4.10 Å, indicating the presence of an L_β’_ phase with pseudohexagonal packing and phospholipid chains tilted with respect to the normal to the bilayer plane [36]. At 15 °C (above the pretransition but below the main transition), there is a peak at 4.15 Å, indicative of a P_β’_ phase. At 30 °C (above the main transition), a very broad peak is observed, which is characteristic of an L_α_ fluid phase.

In the presence of DES, at a 15:1 DMPC/DES molar ratio, the SAXD pattern indicates that this mixture is multilamellar (Figure 4C). The most important difference with respect to pure DMPC was observed at 8 °C because the first order d-spacing increased to 70.89 Å. This first d-spacing obtained from SAXD allows deducing the interlamellar repeat distance, as analyzed below. The effect of DES on this mixture was also reflected in WAXD at 8 °C (Figure 4D), since the observed pattern indicates that the phospholipid chain packing was now in an L_β_ phase in contrast to the L_β’_ observed in pure DMPC at this temperature. In the case of the sample with a 7:1 molar ratio (DMPC/DES), the SAXD diffractograms show patterns similar to those observed for the 15:1 sample (Figure 4E) with an increase in d-spacing at 8 °C with respect to the decrease in d-spacing at 30 °C for pure DMPC for temperatures below T_c_. The pattern of the WAXD was similar to that of the 15:1 sample (Figure 4E).

**Figure 5:**
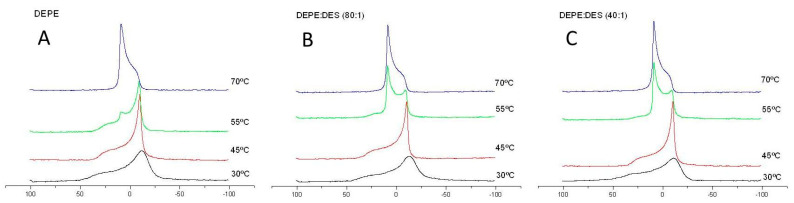
^31^P-NMR spectra of pure DEPE (**A**)) and DEPE containing 80:1 (**B**) and 40:1 (**C**) DEPE/DES molar ratios at different temperatures. The spectra were normalized to the same signal height.

The SAXD diffractograms were further analyzed using the GAP as described in Section 2 in order to investigate the changes in structure occasioned by the incorporation of DES into the DMPC membrane. Note that the first d-spacing order corresponds to the interlamellar repetition distance, which is the sum of the membrane thickness and the water layer separating two membrane layers. Figure 5 and Table 2 show that a change in d-spacing appeared when comparing membranes below and above the phase transition in pure DMPC. This increase was also observed for samples containing DES, and an increase in d-spacing was also observed upon incorporation of DES, with this increase being bigger at temperatures below the phase transition than at temperatures above. Nevertheless, it is remarkable that, in all cases, the increase in d-spacing was due to an increase in dw, i.e., the water layer. In fact, when looking at dHH and dB, it can be concluded that an increase in both temperature and DES concentration produced a small decrease in the thickness of the membrane, which, in the case of dHH, was 3 Å when going from 8 °C to 30 °C for pure DMPC and only 0.8–1.0 Å as a consequence of the addition of DES. Small increases in σC and small decreases in zH were also observed as a consequence of both the increase in temperature and the addition of DES.

### 3.4. Changes in Phase of DEPE as Observed by SAXD and ^31^P-NMR

DEPE presents a characteristic pattern in SAXD because, despite forming MLVs, it is only possible to observe a single d-spacing in both the L_α_ and the L_β_ phase. It is important to observe that the transition of this lipid to the H_II_ phase produced a characteristic pattern in SAXD. Figure 4G shows that, at 68 °C, pure DEPE and the samples containing an 80:1 DEPE/DES molar ratio (Figure 5B) and 40:1 DEPE/DES molar ratio (Figure 5C) showed d-spacings keeping a 1:$1/\sqrt{3}$:1/2$:1/\sqrt{7}$ ratio, which can be associated with an H_II_ phase [32,34,35]. WAXD diffractograms at 30 °C for these samples show a peak characteristic of an L_β_ phase and a diffuse pattern at 45, 55, and 68 °C, indicative of fluid phases.

**Figure 6:**
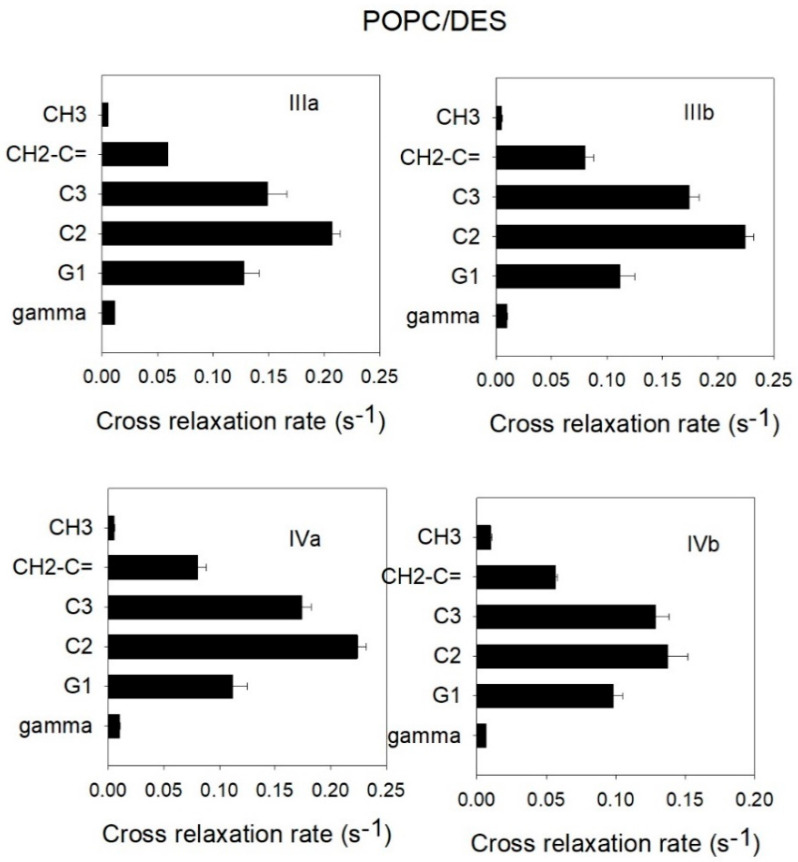
Cross-relaxation rates obtained from the ^1^H-NMR-NOESY spectrum of POPC (1-palmitoyl-2-oleoyl-*sn*-glycero-3-phosphocholine)/DES. Cross-relaxation rates corresponding to the protons bound to the different POPC groups along the long axis of the molecule from the center of the membrane to the polar group (shown in ordinates) with respect to the DES carbons identified in each panel (see Figure 1 to identify the different DES protons studied). Mean values ± standard deviations (five determinations).

The transitions from lamellar to H_II_ phase were also studied by anisotropic ^31^P-NMR [37]. Figure 6 shows that, at 70 °C, pure DEPE and DEPE/DES (80:1 and 40:1 molar ratios) presented a pattern of spectrum with a peak at low field and a shoulder at high field, indicative of an H_II_ phase, whereas, at low temperature (30 and 40 °C), the peak was located at high field and the shoulder of the anisotropic peak was found at low field of the spectrum. It should be remarked that, at 55 °C, there seemed to be a coexistence of lamellar L_a_ and H_II_ phases with two peaks in the samples containing DES.

### 3.5. ^1^H-NOESY-MAS NMR

In order to study the location of the molecule of DES in the phospholipid bilayer, we used ^1^H-NOESY-MAS-NMR. Figure S1 (Supplementary Materials) shows the one-dimensional (1D) spectrum of a POPC/DES (7:1 molar ratio) sample where the main resonance peaks of both POPC and DES (I to IV) were assigned (see Figure 1 to identify protons I to IV). The assignations were made after COSY experiments and integration experiments, according to the literature [38].

On the other hand, 2D-NOESY (Figure S2, Supplementary Materials) allows the measurement of the probability of contact between protons located in different molecules, in this case, between protons located in the phospholipid molecules and in DES. In this way, it is possible to determine the profile of distribution of molecules such as DES.

**Figure 7:**
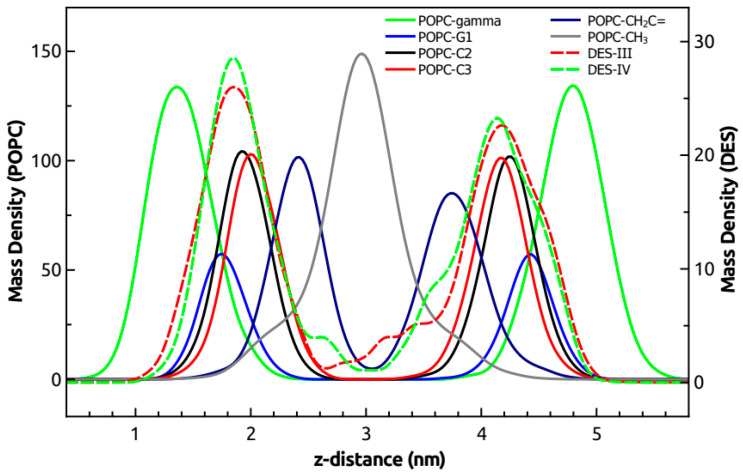
Mass density profiles along the *z*-axis of the membrane (normal to the bilayer) are shown for the gamma (green), G1 (blue), C2 (black), C3 (red), CH_2_C = (dark blue), and methyl terminal (gray) groups of POPC (continuous lines and left axis) and the III (red) and IV (green) groups of DES (dashed lines and right axis). Mass density units are in kg·m^−3^.

To reach this objective, the cross-relaxation rates were calculated, giving a measure of the probability of the contact between two interacting spins. It has been shown that, when this technique is applied to phospholipid membrane systems, it gives information about intermolecular interactions rather than intramolecular ones [14,15]. In consequence, a high value of cross-relaxation rate (s) indicates a high frequency of contact. According to this approach, the most probable location of DES in a POPC bilayer can be deduced. Figure 7 shows that a correlation was established for protons III and IV belonging to DES (Figure 1) and protons clearly assignable to POPC. Protons I and II of DES were not used due to them overlapping with protons of POPC. It can be seen that, in all the cases, the protons from DES had the highest probability of contact with protons bound to carbons C2 and C3 from the fatty acyl chains of POPC, indicating that the DES molecule lies within the part of the phospholipid palisade near the lipid–water interface. It can be concluded that the most likely orientation of DES is with its main axis parallel to the membrane surface. However, other contacts with DES molecules are possible, as can be seen in Figure 7, and this gives a dynamic image of the membrane, as should be expected for a fluid bilayer.

### 3.6. Molecular Dynamics Simulations

To address the structure and dynamics of different types of molecules in relation to their interaction with phospholipid membranes, molecular dynamics simulations have proven to be very useful [39,40] . In this work, molecular dynamics simulations were used to obtain additional evidence to support the experimental results from NMR on the location of DES molecules in the POPC bilayer.

The averaged mass density along the *z*-axis of the simulated system, i.e., the direction normal to the membrane surface, is shown in Figure 7 for the same groups used for cross-relaxation rates obtained from the ^1^H-NMR-NOESY spectrum (Figure 6). The methyl terminal groups of POPC were set as the center of the bilayer, whereas the gamma group indicated the position of the polar headgroup of POPC. DES groups (III and IV) overlapped well with the C2, C3, and G1 groups of POPC, showing a partial overlap with the gamma group of POPC, indicating that DES is mainly located in the hydrophobic region of the membrane but with some interactions with the polar headgroup of POPC. It can also be observed that a small proportion of DES was found in the center of the membrane. These results are in good agreement with the results of cross-relaxation rates obtained from the ^1^H-NMR-NOESY spectrum (Figure 6), where DES was described to have contacts mainly with C2, C3, and G1 and to a minor extent with the methyl and gamma groups of POPC.

**Figure 8:**
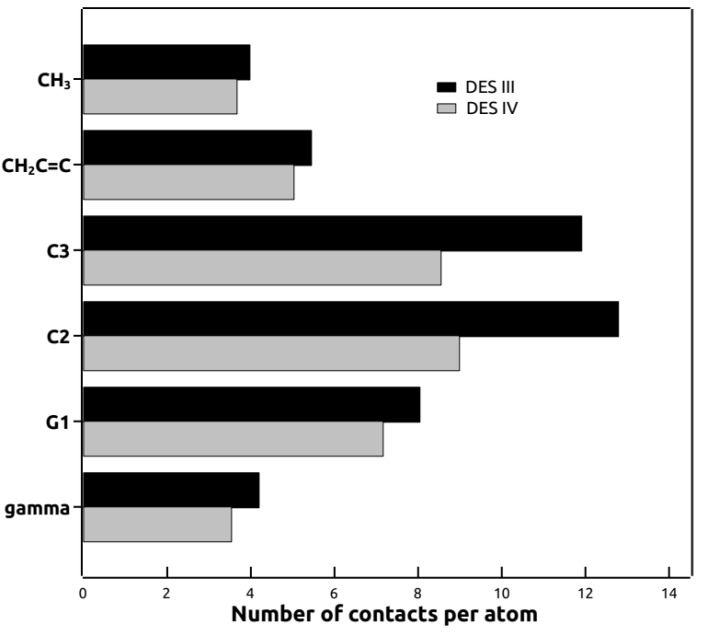
Number of contacts between selected atoms from DES and selected atoms from POPC calculated with mindist function of GROMACS for a trajectory of 60 ns. DES molecules were mainly found close to the polar interface of the bilayer, although a few of them could also be found in the hydrophobic core of the membrane, as also seen in the mass density profiles (Figure 7).

The cross-relaxation rates obtained from NMR experiments (Figure 6) were used as a measured of the distance separating different groups of POPC and DES molecules. As an attempt to reproduce those results using molecular dynamics, the number of contacts between the same atom groups used in NMR was calculated within 0.5 nm, a distance that would include most type of interactions (Figure 8). The number of contacts was calculated as a measure of the proximity of POPC and DES groups for comparison with the NMR results shown in Figure 6. A similar result can be observed from the molecular dynamics distances (Figure 8) to that of NMR (Figure 6), where DES was found to be mainly located near the C2 and C3 groups of POPC.

## 4. Discussion

In this work, we used three different phospholipids, namely, DMPC, DEPE, and POPC. A DMPC membrane was used as a membrane model that undergoes a very cooperative phase transition with a pretransition; from the effects of these transitions on the intrinsic molecules incorporated in the membrane, it was possible to deduce information about the way in which this molecule was disposed in the bilayer. A DEPE membrane has the characteristic of showing a transition from a fluid L_α_ phase to a hexagonal H_II_ phase, and it has been widely used to characterize if a given molecule will have the tendency to facilitate the formation of nonlamellar structures and to induce negative curvature in the membrane [31,41,42]. A POPC membrane was used here as a membrane matrix for the location of DES molecule in the membrane by using 2D-NOESY ^1^H-MAS-NMR; the advantage of using this phospholipid is that it possesses a double bond in the oleoyl chain, and this offers a reference point in the middle of the monolayer, whereas saturated fatty acyl chains do not have this advantage, whereby only protons bound to carbons 2 and 3 and to the last carbon can be differentiated. Furthermore, POPC is fluid at room temperature, which is not the case for saturated chains such as those of DMPC and DPPC. NMR signals need mobility for a convenient resolution; however, measurements at relatively high temperatures are not convenient. Thus, in the case of DMPC, it would be necessary to measure at 30 °C to be well above the transition, equivalent to well above 41.5 °C in the case of DPPC.

It is of interest to discuss the concentrations of DES used in this study. A possibility is to use concentrations similar to those found in the organism, but we did not find this information in the literature. In this type of biophysical study, the limitation is the relatively low sensitivity of the techniques used, and one needs to study samples in which the concentration of DES (in this case) might be higher than that probably found in the organism; however, due to the hydrophobicity of DES, it probably accumulates with time in the membrane after several doses. This uncertainty is a limitation of this study if one wants to extrapolate these results to a situation in vivo.

The interaction of DES with DMPC membranes revealed that DES is incorporated into the membrane, which is indicated by the disappearance of the pretransition, even at low concentrations, and by the decrease in cooperativity of the main phase transition. Interestingly, at higher concentrations of DES, fluid immiscibilities appeared. Fluid immiscibilities originating from the incorporation of intrinsic molecules into membranes were observed previously from DSC experiments, e.g., vitamin K [43,44] and vitamin E [45].

The interaction with DEPE membranes showed that DES decreased the T_c_ transition temperature of DEPE from L_β_ to L_α_. If a molecule is able to incorporate into a membrane, in an intrinsic mode, a lower T_c_ is usually taken as the effect of a contaminant of the phospholipid membrane [46]. On the other hand, molecules that reduce the hydration of the membrane lipid–water interface usually increase T_c_ although they lower T_H_ [47]. As observed by SAXD on DMPC samples, DES increased the hydration of the membranes, as well as favored the transition of the lamellar L_α_ to hexagonal H_II_ phase of DEPE and, hence, induced negative curvature in the membrane. These alterations may be important to understand the effect of DES on the activity of membrane enzymes, since they may alter the interaction of these proteins with the surrounding phospholipids. In a previous paper, it was concluded that the effect of DES as a potent inhibitor of the Ca^2+^-ATPase activity of the sarcoplasmic reticulum is most likely related to the slow conformational transition of the enzyme throughout its catalytic cycle, during which Ca^2+^ is bound at the cytoplasm and dissociated inside the reticulum [8]. DES was found, by means of using n-AS (n-9-anthroyloxy-stearic acid) probes, to be located close to the membrane surface, with this location related to the inhibitory effect in contrast to other similar drugs located at a deeper position in the membrane [8].

The location of DES in the membrane was studied in this work by means of 2D-NOESY ^1^H-NMR-MAS. This technique has been widely used to locate small molecules in phospholipid membranes [48], and, in our laboratory, it was applied to determine the locations of capsaicin [49], a-tocopherol [50,51], and vitamin K [43], among others. This technique has the advantage of directly detecting protons of the studied molecule and not relying on indirect effects such as those on the phospholipids of the membrane. Although high concentrations of the studied molecules are necessary, as is often the case with NMR techniques, the advantage of this technique to study the location of small molecules in phospholipid membranes is clear. With respect to DES, the conclusion from this NMR study is that the most likely location of this molecule in POPC membranes is within the membrane palisade but close to the bilayer surface, with its main axis parallel to the membrane surface.

DES has some similarities to curcumin, since both molecules have a symmetric structure with aromatic rings, and the hydroxyl rings can be expected to anchor both molecules to the lipid–water interface. In fact, this was the location reported recently for curcumin in POPC membranes by using 2D-NOESY 1H-MAS-NMR and molecular dynamics simulations [52]. Similar results to these reported here for DMPC/DES were also obtained from DSC experiments in the mentioned paper with DPPC/curcumin, with the disappearance of the pretransition at low concentrations of curcumin and the appearance of fluid immiscibilities.

The steroid hormone estradiol also shows in its structure two hydroxyl groups, one at each end of the molecule. Its position in the membrane was studied by using NMR techniques and molecular dynamics [53]. It can be expected that both hydroxyl groups would anchor this molecule close to the lipid–water interface, similarly to what we propose for DES. In fact, it was observed that the predominant disposition of estradiol was with the main axis of the molecule perpendicular to the normal to the membrane surface, in full agreement with what could be expected. The steroid hormone 17-β-estradiol was also studied incorporated into DPPC membranes by using DSC, and the authors observed that the characteristic phase transition temperatures of the phospholipid were decreased by the hormone, as detected by DSC [54].

**Figure 9:**
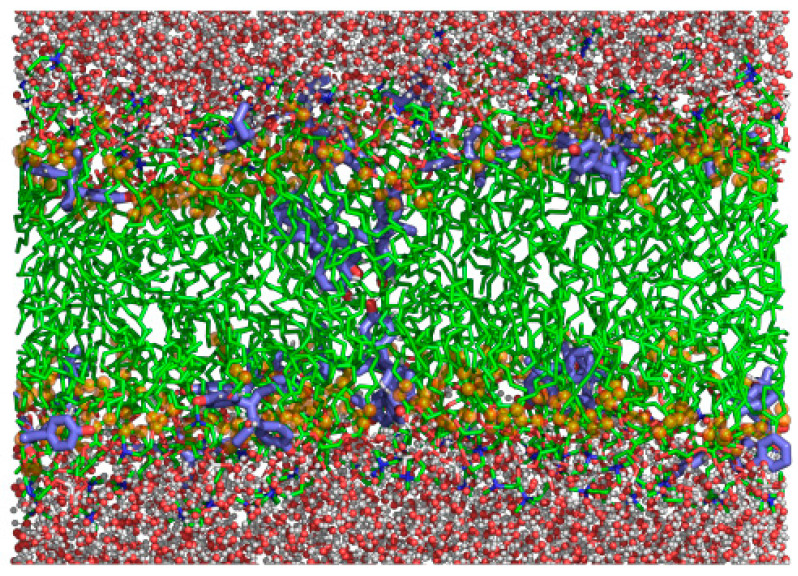
Representative snapshot of POPC bilayer with DES. Water molecules are depicted as balls and sticks, the POPC carbon backbone is shown as green sticks, and the DES carbon backbone is shown as blue sticks. Oxygen atoms are shown in red, and hydrogen atoms are shown in white. The C2 and C3 groups of POPC are shown as brown spheres.

The results obtained after these experiments are in good agreement with those of molecular dynamics simulations where DES was described to have contacts mainly with C2 and C3, and to some extent with the methyl and gamma groups of POPC and this can be also seen in the representative snapshots of a POPC/DES membrane depicted in Figure 9.

This deduction closely agrees with the study carried out previously in sarcoplasmic reticulum membranes using fluorescence quenching by n-AS (n-9-anthroyloxy-stearic acid), with the probe bound at different carbons of the chain [8]; therefore, DES occupies a position close to the Ca^2+^-binding sites of the sarcoplasmic reticulum Ca^2+^-ATPase. The interference with Ca^2+^-binding may explain the inhibitory mechanism observed. In the same way, DES may inhibit the ATPase activity of mitochondrial ATP-synthase [6] by interfering with the coordination between F_o_ and F_1_, which takes place at the membrane surface where this inhibitor is located.

## 5. Conclusions

Our study used different techniques, namely, DSC, X-ray diffraction, NMR, and molecular dynamics simulations. DSC showed that DES disorders DMPC membranes and has poor solubility in this membrane, producing fluid immiscibilities. NMR studies of DES in POPC suggested that the predominant location is within the hydrophobic part of the bilayer but close to the surface, with good proximity to carbons C2 and C3 of these fatty acyl chains. At the same time, experiments with DEPE suggested that DES favors the transition of the lamellar L_α_ to hexagonal H_II_ phase of this phospholipid, indicating its ability to alter the structure of the surface of the membrane. This ability was also indicated by X-ray diffraction studies, which showed an increase in the water layer between different lamellas.

## Figures and Tables

**Table 1:** Changes in ΔH after the addition of DES to membranes of DMPC and DEPE (1,2-dielaidoyl-*sn*-glycero-3-phosphoethanolamine).

Molar Ratio	DMPC/DES
	ΔH (kcal/mol)
1:0	6.5812
60:1	6.3835
30:1	6.3749
15:1	6.2677
7:1	6.198
3:1	5.4762
1:1	4.775
1:0	7.1094
80:1	6.8634
40:1	6.5521
20:1	4.5866
10:1	2.1381

**Table 2:** Parameters extracted from the SADX diffractograms by using the GAP (global analysis program): zH, position of the headgroup Gaussian of the electron density profile; σC, standard variation (width) of the Gaussian describing the hydrocarbon tails; dHH, headgroup peak−peak distance; dB, total bilayer thickness (polar head plus hydrophobic layer); dw, water layer.

	d (Å)	zH (Å)	σC (Å)	dHH (Å)	dB (Å)	dw (Å)
DMPC (8 °C)	64.0 ± 0.2	19.5 ± 0.2	5.4 ± 0.2	39.0 ± 0.3	51.0 ± 1.2	13.0 ± 1.4
DMPC/DES (15:1) 8 °C	70.9 ± 0.2	19.2 ± 0.1	5.9 ± 0.2	38.4 ± 0.2	50.4 ± 1.2	20.5 ± 1.3
DMPC/DES (7:1) 8 °C	72.8 ± 0.2	19.1 ± 0.2	6.1 ± 0.3	38.2 ± 0.3	50.2 ± 1.3	22.6 ± 1.5
DMPC (30 °C)	64.6 ± 0.2	18.0 ± 0.2	6.3 ± 0.2	36.0 ± 0.3	48.0 ± 1.2	16.6 ± 1.4
DMPC/DES (15:1) (30 °C)	65.6 ± 0.1	17.5 ± 0.2	6.4 ± 0.2	35.0 ± 0.3	47.0 ± 1.2	18.6 ± 1.3
DMPC/DES (7:1) (30 °C)	66.7 ± 0.1	17.5 ± 0.1	6.4 ± 0.1	35.0 ± 0.2	47.0 ± 1.1	19.7 ± 1.2

## Data Availability

Data is contained within the article or supplementary material.

## Supplementary Materials

The following are available online at https://www.mdpi.com/2218-273X/11/2/220/s1, Figure S1: 1H-MAS NMR spectra of POPC/DES mixtures, Figure S2: 1H-MAS NMR NOESY spectrum of a POPC/DES sample.
