# Supplementary material for: Diethylstilbestrol Modifies the Structure of Model Membranes and Is Localized Close to the First Carbons of the Fatty Acyl Chains
Source: Biomolecules. 2021 Feb 4;11(2):220. doi: 10.3390/biom11020220 (PMC7914449; doi:10.3390/biom11020220)
Supplement: Supplementary file 1 [file biomolecules-11-00220-s001.pdf]

Supplementary Material

Diethylstilbestrol disorders model membranes induces negative curvature and is localized close to the first carbons of the fatty acyl chains.

Alessio Ausili, Inés Rodríguez-González, Alejandro Torrecillas, José A. Teruel and Juan C. Gómez-Fernández*

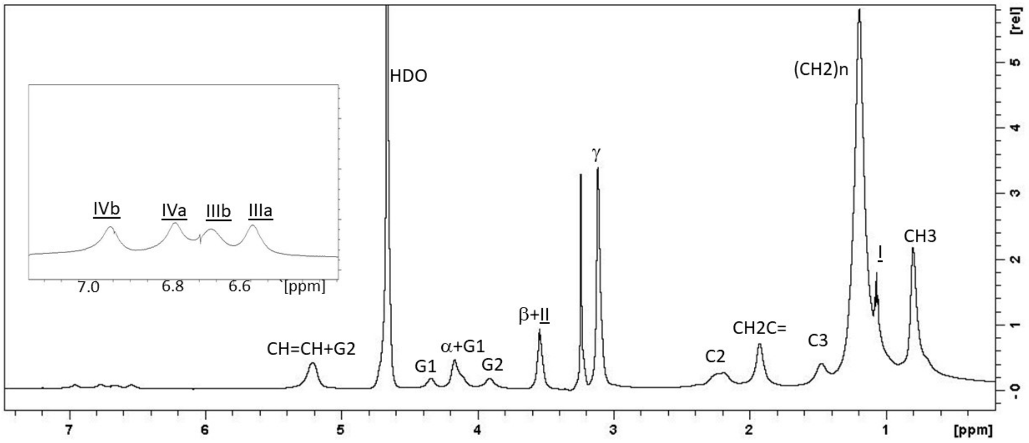

Figure. S1. ^{1}H-MAS NMR spectra of POPC/DES mixtures. Molar ratio was 7:1 phospholipid/DES. Temperature was 25 ºC. The identification of the resonances was done according to the nomenclature for the different carbons used in Fig. 1. Note that I, II, III and IV numbers are used for DES carbons.

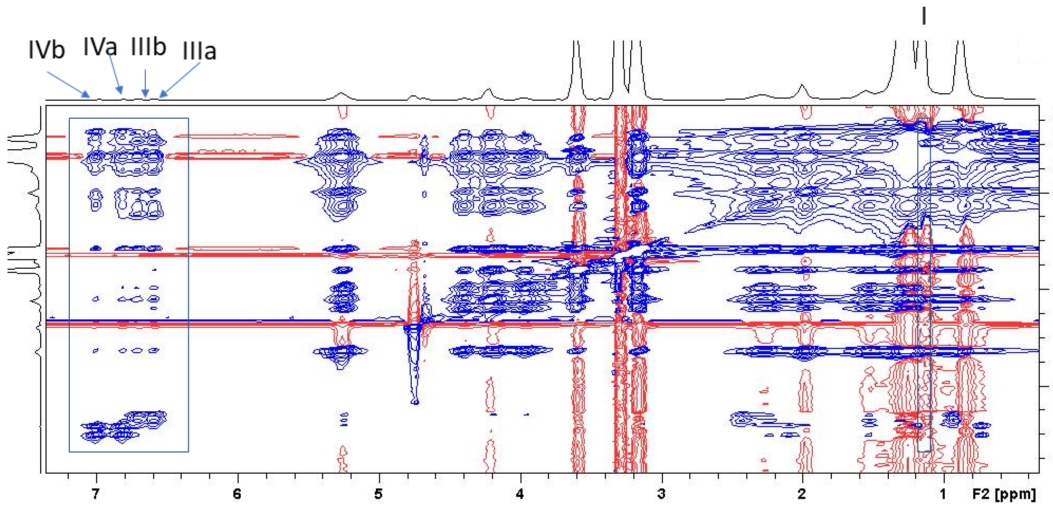

Figure. S2. ^{1}H-MAS NMR NOESY spectrum of a POPC/DES sample. Molar ratio was 7:1 phospholipid/DES. Temperature was 25 ºC. The spectrum was obtained at a mixing time of 300 ms. I, II, III and IV are used to designate the protons bound to carbons of DES, as shown in Fig. 1. The studied cross-peaks are within the framing.
